# A Novel *Ex Vivo* Model to Investigate the Underlying Mechanisms in Alzheimer’s Disease

**DOI:** 10.3389/fncel.2017.00291

**Published:** 2017-09-20

**Authors:** Emanuele Brai, Skye Stuart, Antoine-Scott Badin, Susan A. Greenfield

**Affiliations:** ^1^Neuro-Bio Ltd., Culham Science Centre Abingdon, United Kingdom; ^2^School of Physiology, Pharmacology and Neuroscience, Faculty of Biomedical Sciences, University of Bristol Bristol, United Kingdom

**Keywords:** Alzheimer’s disease, basal forebrain, *ex vivo* brain slices, AChE-peptide, α7-nAChR, p-Tau, Aβ

## Abstract

Currently there is no widely accepted animal model reproducing the full pathological profile of Alzheimer’s disease (AD), since the basic mechanisms of neurodegeneration are still poorly understood. We have proposed that the interaction between the α7 nicotinic acetylcholine receptor (α7-nAChR) and a recently discovered toxic peptide, cleaved from the acetylcholinesterase (AChE) C-terminus, could account for the aberrant processes occurring in AD. In this article we describe a new application on *ex vivo* model procedure, which combines the advantages of both *in vivo* and *in vitro* preparations, to study the effects of the AChE-derived peptide on the rat basal forebrain (BF). Western blot analysis showed that the levels of α7-nAChR, p-Tau and Aβ are differentially expressed upon the AChE-peptide administration, in a selective site-dependent manner. In conclusion, this methodology demonstrates the action of a novel peptide in triggering an AD-like phenotype and proposes a new *ex vivo* approach for manipulating and monitoring neurochemical processes contributing to neurodegeneration, in a time-dependent and site-specific manner.

## Introduction

Alzheimer’s disease (AD) is the most common form of dementia, but the primary events promoting this disorder remain still unidentified. The most popular “amyloid hypothesis” is now being increasingly challenged (Herrup, 2015; Small and Greenfield, 2015; De Strooper and Karran, 2016) and an alternative theory compatible with all clinical features is needed. A new hypothesis suggests that the basic mechanisms of neurodegeneration may occur in a group of neurons which are specifically and primarily vulnerable in AD (Arendt et al., 1992, 2015; Auld et al., 2002; Mesulam, 2004; Schliebs and Arendt, 2011; Schmitz et al., 2016). This heterogeneous neuronal network constitutes a continuous hub of adjacent cell groups, which extend from the basal forebrain (BF) to midbrain and brainstem, areas projecting to key brain areas such as cortex, hippocampus and olfactory bulb (Mesulam et al., 1983). Although this core of susceptible cells display a variety of transmitters, they share the expression of the enzyme acetylcholinesterase (AChE), now established to exert a non-cholinergic function (Greenfield, 2013; Garcia-Ratés et al., 2016). This non-enzymatic role consists in modulating calcium influx into neurons (Greenfield, 2013; Garcia-Ratés et al., 2016) and hence it has trophic or toxic actions depending on dose, availability and neuronal age. In AD therefore, cell death could be attributable to this non-hydrolytic action (Greenfield and Vaux, 2002; Greenfield, 2013; Garcia-Ratés et al., 2016), more specifically via a 14mer peptide cleaved from the AChE C-terminus and included within a more stable, larger peptide, T30 (Greenfield and Vaux, 2002). In addition, the AChE-derived peptide shares a sequence homology with Aβ (Greenfield, 2013; Garcia-Ratés et al., 2016). In AD brains, its concentration is doubled and in PC12 cell lines drives the production of p-Tau and Aβ (Garcia-Ratés et al., 2016) via its binding to the α7 nicotinic acetylcholine receptor (α7-nAChR) and consequent modulation of calcium entry (Greenfield and Vaux, 2002; Greenfield et al., 2004; Bond et al., 2009; Garcia-Ratés et al., 2016). Furthermore, this nicotinic receptor is upregulated upon T30 application in cell cultures (Bond et al., 2009).

Thus, the aims of this work are: (1) describe a novel approach using *ex vivo* brain slices to investigate the early stages occurring during neurodegeneration in intact tissue, whilst maintaining the local neuronal circuitry of the appropriate region and providing the possibility of monitoring acute responses; and (2) evaluate the effects of T30 in a more physiological scenario, *ex vivo* rat brain slices, analyzing its impact on α7-nAChR, p-Tau and Aβ expression. T30 has been applied at a concentration of 2 μM, since this dose was previously administered on the same BF containing sections showing to reduce the neuronal activity via optical imaging experiments (Badin et al., 2016). This methodology could be applied to study diverse molecular cascades, to test pharmacological compounds and provide a reliable tool for drug screening, thereby reducing the number of experiments on living animals.

## Materials and Equipments

### Animals

Nine wild type P14 male Wistar rats were used for this study. This work was performed following the experimental procedures approved by the Home Office (UK) in the guidance on the operation of the Animals (Scientific Procedures) Act 1986 and European guidelines on animal experimentation. The animals were sacrificed respecting Schedule 1 methods (humane killing methods) listed in the Act, which covers all elements of animal research in the UK.

### Reagents for Artificial Cerebrospinal Fluids (aCSFs) Preparation

- Sodium chloride (NaCl; Sigma-Aldrich, S7653, Germany)
- Potassium chloride (KCl; Sigma-Aldrich, P9333, Germany)
- Sodium bicarbonate (NaHCO_3_; Sigma-Aldrich, S5761, Germany)
- Magnesium sulfate heptahydrate (MgSO_4_ (7H_2_O); Sigma-Aldrich, 63138, Germany)
- Potassium phosphate monobasic (KH_2_PO_4_; Sigma-Aldrich, P5655, Germany)
- Hepes salt (Sigma-Aldrich, H7006, Germany)
- Hepes acid (Sigma-Aldrich, H3375, Germany)
- Glucose (Sigma-Aldrich, G7528, Germany)
- Calcium chloride dehydrate (Sigma-Aldrich, 223506, Germany)
- T30 (Genosphere Biotechnologies, France)

These reagents are used to obtain the stock solutions 5× Krebs, 5× Hepes and Sodium bicarbonate in order to prepare the “slicing” aCSF and the “recording” aCSF.

The final concentrations, in mmol, of the two aCSFs (previously described from Badin et al., 2013) are for the “slicing” aCSF: 120 NaCl, 5 KCl, 20 NaHCO_3_, 2.4 CaCl_2_, 2 MgSO_4_, 1.2 KH_2_PO_4_ and 10 glucose; 6.7 HEPES salt and 3.3 HEPES acid; pH: 7.1. “Recording” aCSF: 124 NaCl, 3.7 KCl, 26 NaHCO_3_, 2 CaCl_2_, 1.3 MgSO_4_, 1.3 KH_2_PO_4_ and 10 glucose; pH: 7.1.

### Equipments and Reagents for Brain Dissection, Slicing, Incubation and Homogenization

- Plastic box
- Isoflurane (Henry Schein, cat# 200–070, USA)
- Guillotine
- aCSFs
- Surgical dissecting kit (World Precision Instruments, Item#: MOUSEKIT, USA)
- Surgical blades (Swann-Morton, BS 2982, UK)
- Spatula
- Glue
- Filter paper (Fisher, cat#11566873, USA)
- Vibratome (Leica, VT1000 S, Germany)
- Brushes
- Icebox
- Oxygen canister
- Apparatus (see “Materials and Procedure for the Apparatus Building” Below)
- 1× Phosphate buffer saline (PBS, Fisher, cat# BP2438-4, USA)
- Phosphatase inhibitors (Fisher, cat# 1284-1650, USA)
- Protease inhibitors (Roche complete PIC, 04693116001, USA)
- Pestles (Starlab, cat# I1415-5390, UK)
- Microcentrifuge tubes 1.5–2 ml

### Materials and Procedure for the Apparatus Building

1. Plastic box
2. Air flow control valves (Altec Products Ltd., UK).
3. 2 bundling ties (Altec Products Ltd., UK).
4. Three different sizes PVC tubing. One with a 5 mm bore (cat# 01-94-1584), three pieces with a 3 mm bore (cat# 01-94-1580) and six pieces with a 1.5 mm bore (cat# 116-0536-19) (Altec Products Ltd., UK).
5. One straight barbed and three barbed Y connectors (Cole-Parmer, UK)
6. Glue
7. 12 Hypodermic needles (six 21G, green, and six 25G, orange) (BD Microlance, UK)
8. Six 20 mm injection stopper (Adelphi Healthcare Packaging, INJW20RTS, 1071, UK)
9. Six 5 ml Schott tubular glass Injection vials (Adelphi Healthcare Packaging, VC005-20C, UK)
10. Support

**Figure 1:**
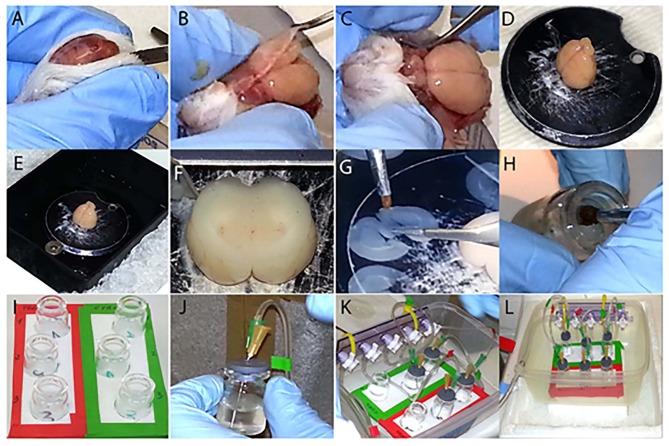
Step by step visualization of the experimental preparation. **(A–C)** Dissection of the brain from the skull. **(D)** Gluing the brain to the vibratome disc. **(E)** The outer buffer-chamber is ice cooled, while the inner part containing the brain is filled with ice cold “slicing” aCSF. **(F)** Sectioning of the brain in coronal slices containing the basal forebrain (BF). **(G)** Division of the slices in two complementary hemisections. **(H)** Disposal of each hemisection in its corresponding vial. **(I)** Three hemisections are transferred into the control group vials (green label), whereas their matching counterparts are placed into the treated group vials (red label). **(J,K)** Each vial is individually sealed **(J)** and placed into the support **(K)**. **(L)** Set up ready for the experiment, connected to the oxygen source and placed on ice.

Fix the (2) to the (1) using (3) (Figure 1K). Connect to one end of the (2) the 5 mm bore tubing (4) which is linked to the oxygen source through the straight barbed connector (5) (Figure 1L). Attach to the lower outlet of the valves the 3 mm bore tubing (Figure 1K). After, insert the three barbed Y connectors (5) in the other side of the 3 mm bore tubing. Attach the small tubes (1.5 mm bore) to each Y shaped connector, in order to have two tubes per each valve (Figure 1K). The free edges of these tubes are glued (6) to the 21G needles (7), which are individually inserted in (8) (Figure 1J), where the 25G needles (7) are also placed. The 25G needles allow the outflow of oxygen excess from (9). Once all the vials (9) (Figure 1I) are closed (Figures 1J,K) position them in the support (10), connect the apparatus to the oxygen source and start the experiment (Figure 1L).

### Western Blot Reagents and Equipment

- Pierce 660 nm Protein Assay (Thermo Scientific, cat# 22660, USA)
- Loading buffer (Bio Rad, cat# 161-0747, USA)
- Beta-mercaptoethanol (Bio Rad, cat# 161-0710, USA)
- Ladder (Bio Rad, cat# 161-0377, USA)
- Running buffer 10× TGS (Fisher, BP1341, cat# 10102823, USA)
- Transfer Buffer 10×, prepared dissolving Tris Base (Fisher Scientific, BP152-500, USA) and Glycine (Fisher Scientific, BP381-500, USA) in distilled water.
- Tris Buffer Saline, TBS 10× prepared dissolving Tris Base (Fisher Scientific, BP152-500, USA) and sodium chloride (Sigma-Aldrich, S7653, Germany) in distilled water.
- Tween 20 (Sigma-Aldrich, P9416, Germany)
- Filter paper (Bio Rad, cat# 1703955, USA)
- PVDF (Immobilon-P) transfer membranes (Sigma-Aldrich, P2563, Germany)
- 4%–20% Mini-PROTEAN TGX Precast Protein Gels (Bio Rad, cat# 456-1094, USA)
- BLOT-FastStain (G-Biosciences, cat# 786-34, USA)
- Blotting grade blocker (Bio Rad, cat# 170-6404, USA)
- Mouse anti-phosphorylated Tau (Thermo Fisher, MN1020, UK)
- Mouse anti-Amyloid beta (Absolute Antibody, Ab00239-2.0, UK)
- Rabbit anti-α7-nAchR (Abcam, ab10096, UK)
- Goat anti mouse HRP conjugated (Sigma-Aldrich, A9309, Germany)
- Goat anti rabbit HRP conjugated (Abcam, ab6721, UK)
- Clarity western ECL substrate (Bio Rad, cat# 170-5061, USA)
- G-Box Chemi XT Imaging System (Syngene, UK)

### Buffers

- 1× PBS
- 1× TBS Dilute the stock solution (TBS 10×) in distilled water.
- 1× TBS-Tween 0.05% (TBST) Dilute the stock solution (TBS 10×) in distilled water and add Tween.
- Lysis buffer Dilute in 1× PBS both protein inhibitors 1:100.

## Stepwise Procedures

### Brain Dissection, Slicing and Experimental Procedure

**Figure 2:**
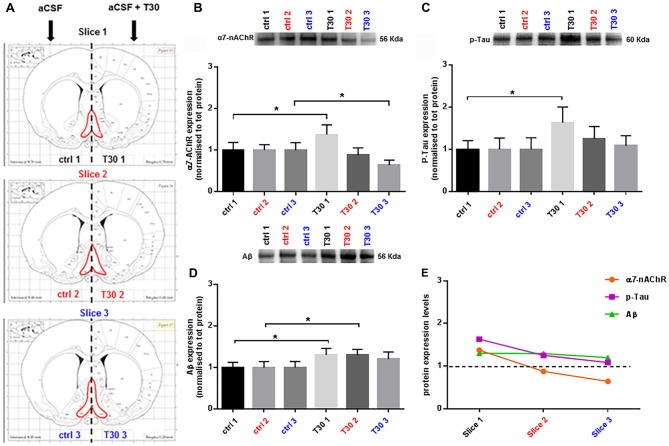
T30 induces a differential expression of α7 nicotinic acetylcholine receptor (α7-nAChR), p-Tau and Aβ. **(A)** Brain atlas coronal slices containing BF (in red), obtained at three anatomical planes of sectioning. **(B–D)** Representative immunoblots and related bar graphs indicating the levels of α7-nAChR **(B)**, p-Tau **(C)** and Aβ **(D)**. **(B)** T30 administration triggers two opposite effects on the α7-nAChR expression, an increase in the rostral section (T30 1) and a reduction in the caudal region (T30 3) compared to the control counterparts (ctrl 1 and ctrl 3). Whereas there is no difference in the intermediate slice (ctrl 2, T30 2). **(C)** p-Tau levels are enhanced in the anterior portion (T30 1) compared to the control matching hemisection (ctrl 1), while no changes between treated and untreated condition are observed in the other two sections (slice 2 and 3). **(D)** Amyloid beta expression is significantly increased in the rostral (T30 1) and intermediate (T30 2) treated hemisections compared to their corresponding control sides (ctrl 2 and ctrl 3), whilst in the caudal slice the two conditions do not show any difference (slice 3). **(E)** Graph summarizing the protein levels of α7-nAChR, p-Tau and Aβ after T30 exposure and compared with the untreated group (black dotted line). The nicotinic receptor and p-Tau display a decreasing pattern, while Aβ has a different trend, showing a constant expression along the antero-posterior axis. Data are analyzed using paired *t* tests and indicated as mean ± SEM. n = (hemislices, rats) is (27, 9). Error bars indicate SEM. *P < 0.05. The images in Panel **(A)** are reproduced with permission from the publisher Paxinos (1998).

Rats were sacrificed as previously described (Badin et al., 2013). Briefly, they were anesthetized with isoflurane, 1.5 ml 100% w/w, poured on the cotton layer inside the induction chamber. After the proper level of anesthesia was confirmed by the absence of the pedal reflex, the animals were decapitated and the brains were quickly and carefully extracted (Figures 1A–C). Shortly, after decapitation the skin covering the skull was cut with a surgical blade and the two sides held under the skull with the index and thumb finger (Figures 1A,B). The skull was subsequently cut along the midline with dissection scissors towards the olfactory bulbs and removed with the forceps retracting the two halves laterally (Figures 1B,C). The brain, once extracted, was cooled and kept hydrated with ice-cold “slicing” aCSF for 1–2 minute (min) and placed on filter paper where the cerebellum was removed with a surgical blade. Finally the brain was vertically glued in the center of the vibratome disc (Figure 1D), placed into the vibratome chamber which was filled with oxygenated ice cold “slicing” aCSF and surrounded by ice (Figure 1E). After regulating the cutting distance (Figure 1F), the brain was sectioned (frequency at 6–7 and speed at 6) in three consecutive coronal sections (300 μm thick) containing BF structures, (approximately Bregma 1.20–0.20 mm; Paxinos, 1998; Figures 1G, 2A). Following the rostro-caudal axis, the sections include the medial septum (MS), diagonal band of Broca (DBB) and, to a lesser extent, the substantia innominata (SI), containing the nucleus basalis of Meynert (NBM). Each slice was subsequently cut along the midline (Figure 1G), in order to obtain two hemisections, used respectively as control and treated preparations at the same anatomical level (Figure 2A). All hemisections were then individually placed in 5 ml glass vials (Figure 1H), where a disc of filter paper was previously inserted to prevent direct contact between the brain tissue and the glass, containing either 3 ml of “recording” aCSF alone for the control group (Figure 1I, green frame) or enriched with 2 μM of T30 for the treated group (Figure 1I, red frame). From top to bottom, the vials in both conditions contained the rostral, the intermediate and the caudal region of the BF (Figure 1I). The hemisections were positioned into their corresponding vials using two different brushes, one for the control group and the other for the treated counterpart, in order to avoid any contamination. Afterwards, the vials were sealed (Figures 1J–L) and carbogen (95% O_2_–5% CO_2_) was continuously provided during the experiment (Figure 1L). The procedure described in Figure 1 was performed within a time window of 10–15 min to minimize any degradation process of the tissue. The oxygen flow was frequently checked, to prevent hypoxia, and kept at the minimum to avoid fluctuation of the hemisections, which could be damaged by the oxygenating needle. After 5 h of incubation, the hemislices were separately transferred, with their respective brushes, into 1.5 ml tubes containing lysis buffer. The brain tissue was then homogenized with pestles, keeping the tubes on ice. To prevent any contamination between samples, each hemisection was processed with a different pestle. The brain lysate was then centrifuged at 1000 *g* for 5 min at 4°C. The supernatant was transferred to a new tube and stored at −80°C until use.

### Western Blot Analysis

Protein concentration was determined with Pierce Assay as previously described (Garcia-Ratés et al., 2016). Aliquots containing 10 μg/μl of proteins were prepared mixing the brain lysate with loading buffer and the denaturing agent beta-mercaptoethanol. Samples were heated at 95°C for 5 min and stored at −80°C until use. Western blot procedure was performed as previously described (Garcia-Ratés et al., 2016). Briefly, proteins were separated through 4%–20% precast gels and blotted on PVDF membranes. After staining the membranes with Blot-FastStain to determine the transfer quality and the total protein content used for the statistical analysis, the blots were destained with warm distilled water until all the bands were removed. Then the blots were blocked in 5% blotting grade blocker in TBS for 1 h at room temperature (RT), with gentle agitation. After the blocking step, the membranes were rinsed 3 × 5 min with TBST and incubated overnight at 4°C with primary antibodies diluted with 1% blocking solution prepared with TBST. The next day, the membranes were thoroughly washed in TBST 5 × 5 min, then incubated for 1 h at RT with the secondary antibodies, diluted in TBST. After the incubation, the blots were rinsed 6 × 5 min with TBST and 10 min with TBS and probed for protein revelation using chemiluminescent substrates. The primary antibodies, all diluted at 1:1000, were: mouse anti-phosphorylated Tau, mouse anti-Amyloid beta and rabbit anti-α7-nAchR. The secondary antibodies, all HRP conjugated, were goat anti mouse (1:2000) and goat anti rabbit (1:5000).

### Statistical Analysis

Protein values were normalized to total protein expression (Supplementary Figure S1; Aldridge et al., 2008; Zeng et al., 2013) and analyzed with ImageJ software (NIH, Bethesda, MD, USA). Differences in protein levels between control and treated group were determined using paired Student’s *t*-test and subsequently plotted with Graphpad software (Graphpad Prism 6, San Diego, CA, USA). All data were considered significant with a *p*-value < 0.05.

### Troubleshooting

Table 1 indicates possible issues which can occur during the procedure, their related cause and suggestions to solve and avoid them.

**Table 1:** Troubleshooting.

Problem	Possible cause	Solution
**Poor quality of the tissue**.	Mechanical damage during brain removal or slicing.	Operate gently and precisely while handling the tissue.
	Slow dissection and slicing.	Practise to dissect out and process faster the brain.
	The tissue is not kept cold.	Ensure that the brain is preserved on ice cold aCSF during removal and sectioning.
	The hemisections are floating inside the vials.	Reduce the oxygen flow and/or move the “oxygenating” needle if too close to the hemislice to avoid a direct contact which can impair the integrity of the tissue.
	Old solutions.	Prepare frequently fresh solutions.
	Anoxia during the experiment.	Check that the slices are constantly oxygenated.
**Absent or inconstant oxygenation**.	The carbogen canister is empty.	Check before starting the experiment that the oxygen is sufficient for all the steps.
	The valves are closed.	Control the canister and in particular the apparatus’ valves, opening them before starting the procedure.
	The tubing are not properly connected or broken.	Check periodically the condition and functionality of the components and change them if damaged.
	The “oxygenating” needle is plugged.	Remove quickly the injection stopper from the vial and flick the needle. A piece of tissue or filter paper could block the oxygen flow.
	The “oxygenating” needle is not immersed in the aCSF.	Ensure that the needle is properly immersed within the aCSF.

### Timing

- Brain removal: within 1 min
- Brain preparation for sectioning (attachment to the vibratome disc and placement inside the slicing chamber): 1–2 min
- Slicing: within 5 min
- Transfer of the hemislices in the vials and starting the experiment: 1–2 min
- (During this step the slices are not oxygenated until all the lids are closed and the apparatus is connected to the oxygen source, so is important to execute it quickly).
- Experiment length: 5 h
- Tissue transfer into the 1.5 ml tubes and homogenization: within 10 min
- Centrifugation of the brain lysate: 5 min
- Transfer of the supernatant into new tubes: 1–2 min
- The time in each step, excluding the 5 h incubation and centrifugation, can vary depending on the experience of the researcher.

### Advantages and Limitations of the Protocol

This protocol provides several advantages: (1) it maintains the anatomical site-selectivity and the physiological milieu of the living brain; (2) slices can be easily obtained for studying a specific area; (3) depending on the characteristics of the investigated region, such as size and discernible boundaries, a microdissection could be performed after the treatment, providing a more precise read out of the investigated area; (4) direct intervention, precision dosing and an untreated contralateral side from the same slice offers an accurate control; and (5) the potential reduction in the number of living animals ultimately used in behavioral studies, based on the ability of multiplexing the desired experimental conditions. The limitations are: (1) the thickness and integrity of the tissue; (2) the incubation time; and (3) severing of connections in the rostro-caudal plane. All these aspects could affect biochemical, molecular or immunohistochemical analysis. However, the integrity of the tissue can be determined through immunohistochemical detection of several markers specific for neuronal viability and electrophysiology techniques as previously described (Cho et al., 2007; Badin et al., 2013).

## Anticipated Results and Discussion

### T30 Treatment Affects α7-nAchR, p-Tau and Aβ Levels in a Site-Specific Manner

After 5 h, T30 exposure (Figure 2A) induced a heterogeneous expression of α7-nAChR (Figure 2B), p-Tau (Figure 2C) and Aβ (Figure 2D) along the BF rostro-caudal axis. The α7 receptor, showed a 38% increase in the rostral portion (slice 1, *p* = 0.0310) in the treated group (Figure 2B), whereas in the intermediate slice there was no difference in the two groups (slice 2, *p* = 0.1195; Figure 2B). In the caudal section its levels were reduced by 36% compared to the control group (slice 3, *p* = 0.0476; Figure 2B). Phosphorylated Tau displayed higher levels, 63%, in the rostral hemisection upon T30 application, (slice 1, *p* = 0.0158; Figure 2C), while the other slices did not show any change between treatments (slice 2, *p* = 0.1014; slice 3, *p* = 0.6405; Figure 2C). Aβ levels were significantly increased by 30% in the anterior and intermediate portion in the treated hemisections (slice 1, *p* = 0.0136; slice 2, *p* = 0.0109), whereas in the posterior slice no difference was observed in the treated side above the control (slice 3, *p* = 0.1231; Figure 2D). Figure 2E recapitulates the expression patterns of the three markers upon T30 administration compared to the control value (black dotted line). Furthermore, along the rostro-caudal plane, the peptide induced two different trends in the protein levels, one promoting a continuous reduction of α7-nAChR and p-Tau and the second displaying a similar Aβ overexpression over the control (Figure 2E).

## Discussion

The essential feature of this procedure is the opportunity to monitor, in a time and site-dependent manner, key neurochemical events potentially underlying neuronal dysfunction in a preparation that combines the advantages of an *in vivo* physiological milieu, with intact brain cytoarchitecture and local synaptic network, with those of *in vitro*, such as precision, accessibility and control of the extracellular environment.

This protocol is an adaptation of the well-established *ex vivo* brain slice model for real-time recordings, widely used for electrophysiology (Sakmann and Neher, 1984; Jensen et al., 1991; Tozzi et al., 2015; Ferrati et al., 2016) or optical imaging (Grinvald and Hildesheim, 2004; Badin et al., 2013, 2016). Despite the different methods where T30 has been previously tested (Badin et al., 2013, 2016; Greenfield, 2013; Garcia-Ratés et al., 2016), we showed for the first time in this work the T30 mediated responses using a novel approach on *ex vivo* brain slices. In this study, our goal was to evaluate the T30 effects on the BF, over hours, in a more physiological context than tissue culture (Bond et al., 2009; Garcia-Ratés et al., 2016) and demonstrate its contribution to slower neuronal processes characterizing the pathophysiology of neurodegenerative disorders. The data presented here indicate that, following T30 administration, a distinct anatomical-specific expression of the three proteins occurs. The nicotinic receptor showed an increase in the rostral treated side and a decrease in the caudal one compared to the untreated group (Figure 2B, slice 1 and 3), whereas the intermediate region does not show any change between the two conditions (Figure 2B, slice 2). These results are consistent with former studies describing a T30 mediated expression of the α7-nAChR (Bond et al., 2009).

Phosphorylated Tau levels were higher only in the rostral treated hemisection compared to the control counterpart (Figure 2C, slice 1), while no variation was observed in the other slices comparing the two conditions (Figure 2C, slices 2 and 3). On the other hand, Aβ was constantly overexpressed in all the T30 exposed hemisections (Figure 2D), showing a significant increase in the rostral and intermediate region above the control levels (Figure 2D, slices 1 and 2). These results suggest that the AChE-peptide could exert a pivotal role in varying the protein expression along the rostro-caudal axis (Table 2), since, in contrast, in all the controls the protein levels are similar (Figures 2B–E). Further observation was the similarity in the expression pattern shown by α7-nAchR and p-Tau, i.e., T30 administration increased their levels in the rostral portion and then a gradual decrease in the other two regions (Figure 2E). Conversely, Aβ levels were similarly enhanced in all the regions compared to the other two markers (Figure 2E).

**Table 2:** Anatomical specificity in protein expression induced by T30 administration.

AD hallmarks	Slice 1 (rostral)		Slice 2 (intermediate)		Slice 3 (caudal)	
	ctrl 1	T30 1	ctrl 2	T30 2	ctrl 3	T30 3
α7-nAChR	1	+	1	=	1	−
p-Tau	1	+	1	=	1	=
Aβ	1	+	1	+	1	=

*The protein levels are shown comparing the amount observed in the treated hemisections with their control counterparts, following the rostro-caudal axis. +, −, = indicate a significant increase, decrease or no change in protein expression in the T30 exposed group, compared to the normalized control values, designated with 1*.

As a consequence, the influx of calcium into each BF sub-division (triggered by AChE-peptide), through a heterogeneous density of the α7-nAChR, may in turn favor p-Tau or Aβ pathways. In any event, our data are in line with earlier evidence indicating a functional interaction involving this receptor with p-Tau and Aβ (Wang et al., 2000; Dineley et al., 2002; Rubio et al., 2006; Oz et al., 2013). Furthermore, several findings indicate that the BF nuclei are not homogenous in their physiological characteristic: they display a diverse neuronal morphology (Dinopoulos et al., 1988), as well as a differential anteroposterior distribution of GSK-3β (Leroy and Brion, 1999), which is indirectly activated by T30 (Bond et al., 2009) and regulates Tau phosphorylation and Aβ formation (Garcia-Ratés et al., 2016). In addition, these molecular mechanisms might mediate cell death, which in AD occurs within the BF in a site specific manner (Arendt et al., 1983; Liu et al., 2015; Schmitz et al., 2016).

In conclusion, we demonstrate that T30 administration on *ex vivo* brain slices containing BF alters, in a site-specific way, the expression of the α7-nAChR, which could contribute to the modulation of p-Tau and Aβ. Noteworthy, it has to be considered that different concentrations of the peptide could determine dose-dependent outcomes, either increasing or decreasing the protein levels compared to the data presented in this work. Beyond the validation of the specific hypothesis explored here, this technique could open up a valuable approach to investigate a wide range of neuropathological processes, since it preserves the cytoarchitecture of the brain region, its local circuitry and the neuronal matrix over a sustained time window, thereby providing a direct and sensitive read-out of a specific level of the brain under physiological and pathological conditions.

## Author Contributions

EB planned and performed the experiments, analyzed the data, wrote the article. SS performed the experiments and reviewed the manuscript. A-SB showed EB the experimental procedure. SAG revised the manuscript and designed the study. All authors read and approved the final manuscript.

## Conflict of Interest Statement

The authors declare competing financial interest. SAG is the founder and CEO of Neuro-Bio Ltd, a privately owned Company and holds shares in the Company. EB and A-SB are employees of the Neuro-Bio Ltd. SS is an undergraduate student at the University of Bristol, on industrial placement with Neuro-Bio for 9 months.

## Supplementary Material

FIGURE S1 Staining of the total protein expression used for statistical analysis. **(A–C)** Representative immunoblots showing the whole protein content used to normalize the levels of α7-nAChR **(A)**, p-Tau **(B)** and Aβ **(C)**.
