# Supplementary figures and images for: A Novel Ex Vivo Model to Investigate the Underlying Mechanisms in Alzheimer’s Disease
Source: Front Cell Neurosci. 2017 Sep 20;11:291. doi: 10.3389/fncel.2017.00291 (PMC5627036; doi:10.3389/fncel.2017.00291)

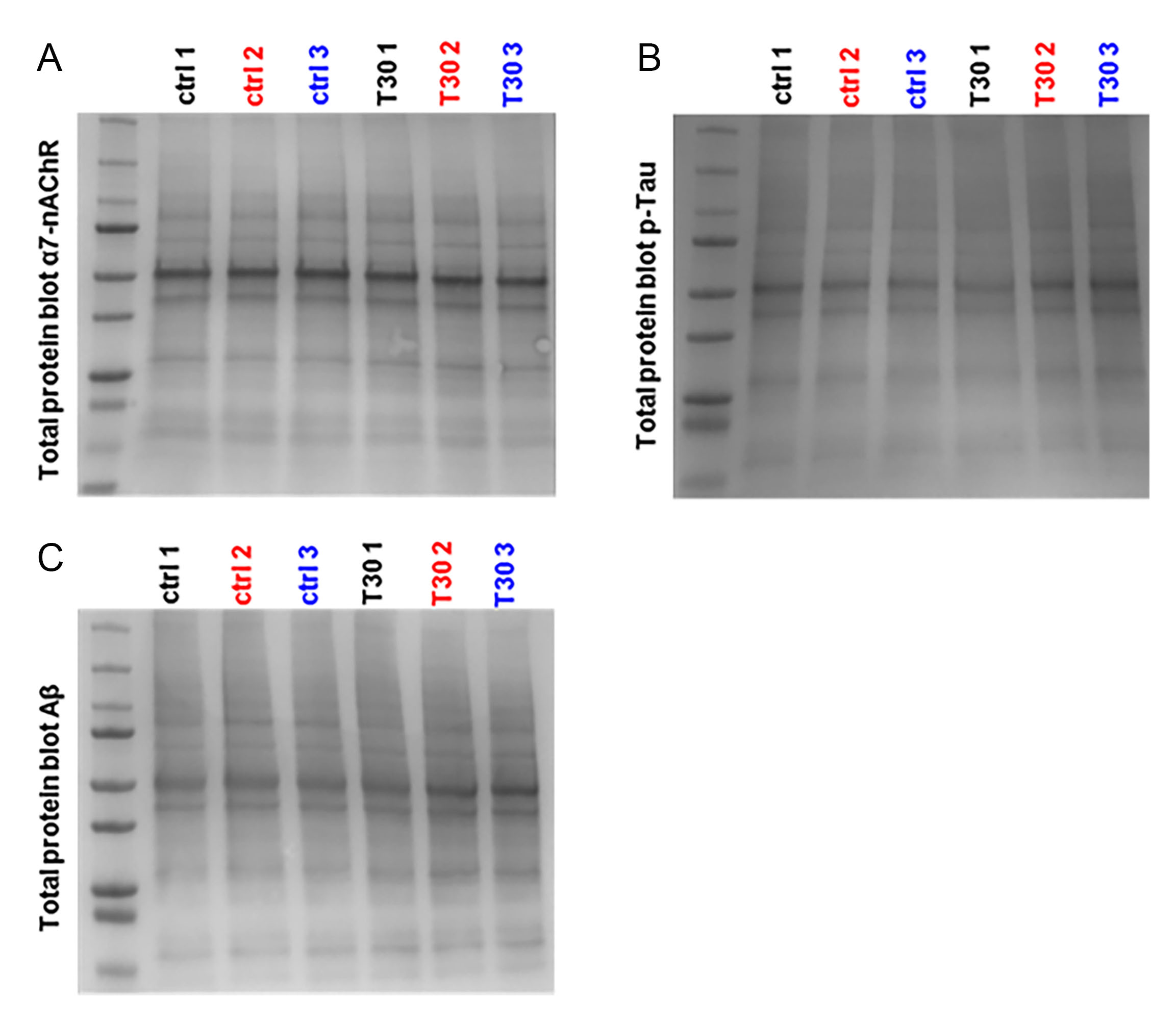

Supplement: FIGURE S1 — Staining of the total protein expression used for statistical analysis. (A–C) Representative immunoblots showing the whole protein content used to normalize the levels of α7-nAChR (A), p-Tau (B) and Aβ (C). [file Image_1.jpeg]
